# Normative data for an expanded set of stimuli for testing high-level influences on object perception: OMEFA-II

**DOI:** 10.1371/journal.pone.0224471

**Published:** 2020-08-14

**Authors:** Colin S. Flowers, Kimberley D. Orsten-Hooge, Barnes G. L. Jannuzi, Mary A. Peterson

**Affiliations:** 1 Department of Psychology, University of Arizona, Tucson, Arizona, United States of America; 2 School of Behavioral and Brain Sciences, The University of Texas at Dallas, Dallas, Texas, United States of America; 3 Neuroscience and Cognitive Science Program, University of Arizona, Tucson, Arizona, United States of America; 4 Cognitive Science Program, University of Arizona, Tucson, Arizona, United States of America; University of Muenster, GERMANY

## Abstract

We present normative data for an expanded set of stimuli designed to investigate past experience effects on object detection. The stimuli are vertically-elongated “bipartite” displays comprising two equal-area regions meeting at an articulated central border. When the central border is assigned to one side, a shaped figure (i.e., an object) is detected on that side. Participants viewing brief masked exposures typically detect figures more often on the *critical* side of *Intact* displays where a common (“familiar”) object is depicted than on a matched critical side of *Part-Rearranged* (*PR*) displays comprising the same parts arranged in novel configurations. This pattern of results showed that past experience in the form of *familiar configuration* rather than *familiar part*s is a prior for figure assignment. Spurred by research implicating a network involving the perirhinal cortex of the medial temporal lobe in these familiar configuration effects, we enlarged the stimulus set from 24 to 48 base stimuli to increase its usefulness for behavioral, neuropsychological, and neuroimaging experiments. We measured the percentage of participants who agreed on a single interpretation for each side of *Intact*, *Upright PR*, and *Inverted PR* displays (144 displays; 288 sides) under long exposure conditions. High inter-subject agreement is taken to operationally define a familiar configuration. This new stimulus set is well-suited to investigate questions concerning how parts and wholes are integrated and how high- and low-level brain areas interact in object detection. This set also allows tests of predictions regarding cross-border competition in figure assignment and assessments of individual differences. The displays, their image statistics, and normative data are available online (https://osf.io/j9kz2/).

## Introduction

A fundamental aspect of visual perception involves detecting where objects lie in a scene. An object is often detected on only one side of a border shared by two regions in the visual field; the other side, lacking a border, is perceived as a locally shapeless back*ground* to the figure (e.g., [1–4]). This outcome, commonly called figure-ground perception, is essentially object detection [5]. Therefore, it is important to understand how it occurs.

It has long been known that figure-ground perception (i.e., object detection) is influenced by properties associated with figures rather than backgrounds. Such *figural priors* include enclosure, symmetry, surroundedness, small area, convexity, and contrast; more recent research showed that lower region and top-bottom polarity are also figural priors (e.g., [4, 6–11]; for reviews: [1, 3, 12]). The aforementioned figural priors are image characteristics; their influence on figure-ground perception can be explained without invoking observers’ past experience. Indeed, it was long held that past experience could not affect figure assignment.

**Fig 1:**
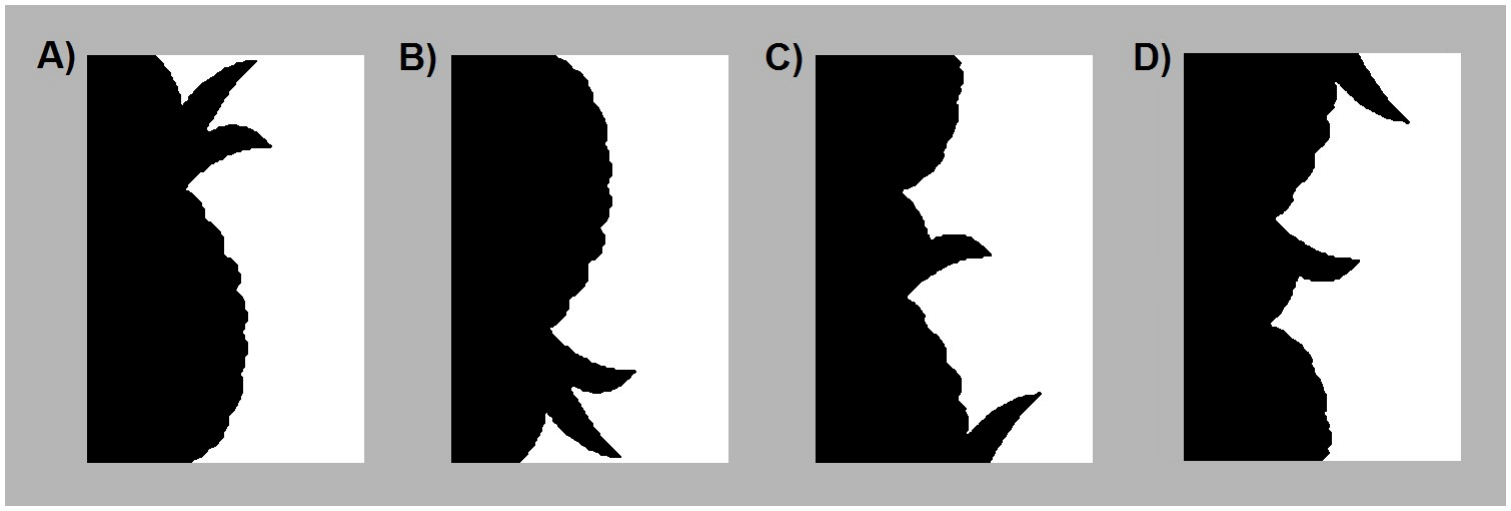
A sample bipartite stimulus in 4 configurations. In this figure, the critical side is presented in black on the left of the central border. When these stimuli are used, black/white contrast and left/right location of the critical side is balanced; they are presented on a medium gray backdrop. A) *Upright Intact*, B) *Inverted Intact*, C) *Upright Part-rearranged*, D) *Inverted Part-Rearranged* versions of the source stimulus, “Pineapple”.

However, strong evidence has accumulated that past experience in the form of *familiar configuration* affects figure assignment: Observers are likely to perceive figures on the side of a border where a portion of a common object is sketched (e.g., [2, 13, 14]; for review: [15, 16]). In many of the experiments demonstrating effects of familiar configuration on figure assignment *bipartite displays* were used (see Fig 1); these are elongated rectangular stimuli divided into two equal-area regions by a central vertically-oriented border that sketches a portion of a common mono-oriented object on one side but not the other. Image characteristics and other known figural priors are matched as closely as possible on the two sides of bipartite displays. Participants report the side of the central border (left / right) on which they perceive the figure. Bipartite displays where potential objects are defined solely by the central border provide a controlled way to measure effects of past experience on object detection.

Past experience effects are not measured as differences in the likelihood of perceiving the figure on the side of the central border where the familiar configuration lies (*the critical side*) versus the opposite side (*the complementary side*). This is because, despite attempts to match the two sides of bipartite displays for image features, some local or global differences may remain. Instead, past experience effects are measured as differences in the likelihood of perceiving the figure on the critical side of the border when the displays are upright such that the common objects are sketched in their familiar upright orientation versus inverted such that the common objects are misoriented from their familiar upright by 180°. An orientation change of 180° holds image-based differences between the critical and complementary sides of the bipartite displays constant while increasing the response time of cell populations signaling the presence of familiar configurations (cf., [17]). Orientation-dependent effects of familiar configuration have been reliably obtained, revealing that past experience is a prior for object detection (e.g., [2, 14, 18, 19]).

The orientation dependency of familiar configuration effects also suggests that there is a short time window after stimulus onset during which past experience must be activated in order to affect figure assignment. It is not clear how long this time window is, but Trujillo et al. [20] and Sanguinetti et al. [21] demonstrated that for upright displays past experience is activated within 110 ms after stimulus onset. Even though familiar configuration doesn’t operate as a figural prior for inverted displays, once critical regions are perceived as figures in inverted displays (either by chance or by intentional reversal of figure assignment), the common objects they depict can be recognized (e.g., [14, 22]; for review see [5]). After object detection, identification of misoriented version of objects with typical uprights is delayed but is not prevented (cf., [23, 24]). Before object detection, however, effects of the figural prior of familiar configuration are absent when objects sketched on the critical side of the border are misoriented from their typical uprights.

The portions of familiar objects sketched by the central borders are schematized. Therefore, it is improbable that experimental observers have encountered the particular configurations tested before; hence, *familiar configuration* effects reveal the influence of past experience with a class of objects (e.g., objects within a basic-level category) rather than with the specific exemplars sketched by the border [25]. In most experiments investigating familiar configuration effects, stimuli are not repeated; though effects of repetition within an experiment have also been demonstrated (e.g., [26–28]) Thus, evidence that familiar configuration is a figural prior demonstrates that high-level object properties abstracted across many previous instances influence object detection.

Additional evidence that high-level processing is involved in familiar configuration effects is that effects of familiarity are mediated by configurations rather than parts: past experience effects are usually not observed in *Part-Rearranged* displays (*PR* displays) in which the critical side of the display sketches a novel configuration created by spatially rearranging the parts of a familiar configuration (e.g., [14, 19, 22, 29]). Barense, et al. [18] recently reported that participants with damage to the perirhinal cortex (PRC) of the medial temporal lobe (MTL) are exceptions, however; these participants perceived the figure on the critical side of *PR* displays as often as on the critical side of displays suggesting intact familiar configurations (*Intact* displays). The PRC is a high-level area long thought to be involved in declarative memory only, yet fMRI and neuropsychological evidence indicates that it also plays a role in visual perception (e.g., [18, 30, 31]). Following Barense et al. [18], fMRI experiments showed that the PRC responds differently to *Upright Intact* displays, *Upright PR* displays, and *Inverted PR* displays and suggested that PRC activity may modulate neural responses in visual area V2 where receptive fields are too small to encompass entire configurations [32, 33]. These results expanded the literature regarding the role of the PRC beyond odd-one-out tasks and tasks requiring stimulus scrutiny into object detection, a fundamental aspect of visual perception. They also raised important questions regarding the role of dynamic interactions across brain areas as high as the MTL and as low as V2 in object detection. Therefore, sets of stimuli designed to test for familiar configuration effects on figure assignment provide an important resource for both vision and memory scientists investigating questions concerning how parts and wholes are integrated and high- and low-level brain areas interact in object detection. Our goal was to provide normative data for an expanded set of stimuli for testing effects of past experience on figure assignment.

### The current normative study

It is difficult to create bipartite stimuli due to the constraints that must be met to examine figure assignment: the two sides of the display must be equal in area; the common objects must have sufficient part structure to allow creation of *PR* displays; and the central border must sketch a common object with a canonical upright orientation that can be identified on the basis of the portion sketched by the border. This last criterion raises the question of how familiar configuration has been operationalized. It is not sufficient for experimenters to agree that a critical region depicts a well-known object. Instead, a group of naïve pilot participants have been asked to view the bipartite displays and while doing so, to perceive each side as figure successively, and to identify any common objects resembled by (denoted by) each side of the schematized central border. High inter-subject agreement has been taken to indicate that the familiar configurations activate past experience, although this operational definition cannot assess how quickly past experience is activated [2, 19].

Previous experiments have used a set of 24 *Intact* display and their *PR* counterparts; the stimulus set was called the Object Memory Effects on Figure Assignment (OMEFA) set. In order to maximize the usefulness of bipartite stimuli for current researchers, we expanded the basic set of bipartite stimuli from 24 to 48 and created three types of displays for each one: *Upright Intact*, *Upright PR*, and *Inverted PR* displays. (Three stimuli from the original set for which novel *PR* displays could not be created–a candle, a stop sign, and the letter F–were replaced.) We obtained normative data in the form of inter-subject agreement regarding resemblance to common objects for 288 regions (i.e., both the critical and the complementary sides of 48 versions of the three types of displays). Inter-subject agreement had not previously been assessed systematically for either *Upright* or *Inverted PR* displays or for the either side of the new stimuli. Since figure assignment entails competition between objects that might be perceived on opposite sides of a border (e.g., [25, 34–38]), it important to assess inter-subject agreement for both sides of bipartite displays. Moreover, with normative data for both sides of bipartite displays, inter-item differences can be examined based either on inter-subject agreement regarding the critical or complementary sides or on the differences in inter-subject agreement regarding the critical and complementary sides.

We did not measure inter-subject agreement regarding *Inverted Intact* displays because our intention is to gather normative data regarding the extent to which the critical and complementary sides of our displays resemble common objects with tops specified in viewer-centered coordinates. As discussed previously, once the critical region is perceived as the figure in *Inverted Intact* displays it can often be identified as depicting an inverted version of common object. Therefore, we reasoned that including *Inverted Intact* displays in the norming study could cause participants to search for interpretations with misoriented tops in all of the displays, hence rendering the normative data unusable. We have observed this tendency previously (e.g., [19]).

We gathered normative data for 27 new bipartite displays and for 21 updated versions of bipartite displays in the original OMEFA set. Therefore, all the normative data for the new set–“OMEFA-II”–are contemporary. Moreover, the borders of the stimuli in the OMEFA-II set are smoother than those of the original OMEFA stimuli; hence, a potential benefit is that there will be less noise in the activation of past experience (cf., [39]). Another benefit of the OMEFA-II stimulus set is that image statistics are provided for each stimulus (see S1 Appendix). This will provide sufficient information about each stimulus so that experimenters can choose which stimuli to include in their experiments and which comparisons to make given their own experimental questions.

We used the Amazon Mechanical Turk (AMT) platform to gather the normative data. Individual participants viewed and responded to stimuli of all three display types, but they saw a stimulus derived from a particular *Intact* stimulus in only one display type. They viewed each stimulus for as long as they wished and listed up to three interpretations for each side of each bipartite display. The viewing distance and, by extension, the size of the stimuli was uncontrolled by virtue of using remote participants through AMT. The data collected are not intended to reflect visual perception mechanisms, but rather to provide normative data about the extent to which the central border suggests common objects under unconstrained viewing. We expected to obtain high inter-subject agreement for the critical sides of many of the *Upright Intact* displays, but not their variants which were intended to control for image features and parts while reducing or eliminating effects of familiar configuration. For objects with distinctive parts, we expected that the parts might support some degree of inter-subject agreement for the critical sides of *PR* displays, although not as much as for the critical sides of *Upright Intact* displays. We note that our method assesses explicit identification of common objects, which we assume is related, but not identical, to implicit activation of past experience which serves as a figural prior.

## Methods

All research was approved by the Human Subjects Protection Program at University of Arizona. Consent was obtained by all participants who had to press a button on the online experiment indicating that they agreed to consent. Furthermore, all data files have participant worker ID numbers deidentified.

### Participants

Potential participants had to meet the eligibility criteria of (a) having completed 1000 tasks or other data collection programs on AMT and (b) having achieved an approval rating of at least 95% (see [40]). A total of 194 AMT participants met these criteria. Responses from 16 of these participants were excluded because they failed attention check trials (see Procedure); responses from four other participants were excluded because they were gibberish or non-words. Responses from the remaining 174 participants were analyzed.

Participants were compensated $1.50 to complete the task. Pilot tests showed that the tasks took no more than 10 minutes to complete (and could be completed much faster). Therefore, the estimated rate of pay was at the very least $9.00 per hour (above the US national minimum of $7.25 when these data were gathered).

### Stimuli

Bipartite displays are vertically elongated displays comprising two regions situated on the left and right sides of a central border. One region is black and the other white; they are presented on a medium gray background such that the black and white regions contrast equally with the background (see Fig 1). Using AMT, we could not control exact luminance values on participants’ screen. We used pixel RGB values of: black = [0 0 0], white = [255 255 255], gray = [182 182 182]. These RGB values yielded luminance values of 0.12, 87.33, and 45.70 fL respectively on the computers in our laboratory. Although the luminance values surely differed for each individual AMT participant, between-subject differences in display luminance would not differentially affect their responses to the different stimulus types.

The two regions of each bipartite display were equated for area by equating the number of pixels in the critical and complementary regions (mean % pixels on the critical side = 49.99% for *Intact* displays and 50.00% for *PR* displays; see S1 Appendix for image characteristics). We tested 48 bipartite displays with critical sides sketching *Upright Intact* portions of 48 well-known objects, 48 *Upright PR* displays–one for each of the *Upright Intact* displays, and 48 *Inverted PR* displays; 288 regions of 144 displays overall. In what follows, we denote the stimuli by the name of the familiar configuration intended to be depicted by the *Upright Intact* displays (the “source” name) modified by display type.

The 144 stimuli tested are listed in Table 1 and can be accessed online (https://osf.io/j9kz2/). Stimuli were 343 pixels high (H) and ranged from 111 to 350 pixels wide (W). AMT participants viewed the stimuli at different viewing distances and on screens with different sizes and different resolutions; hence, stimulus size was not matched across subjects in this study (although it was matched for the different display types for each participant). The number of pixels in the stimuli was large enough that we could be reasonably confident that the stimuli were of sufficiently high resolution under these disparate conditions.

**Table 1:** Percent inter-subject agreement for each side (critical and complementary) and critical–complementary difference scores for three types of OMEFA-II bipartite stimuli: *Upright Intact*, *Upright Part-Rearranged*, and *Inverted Part-Rearranged*.

	**Upright Intact**					**Upright Part-Rearranged**					**Inverted Part-Rearranged**				
**Source**	**Critical**	**%**	**Comp**	**%**	**Diff**	**Critical**	**%**	**Comp**	**%**	**Diff**	**Critical**	**%**	**Comp**	**%**	**Diff**
Lamp	Lamp	100.0	Furniture	18.8	81.3	Keyhole	46.9	Vase	9.4	37.5	Vase	28.1	‘3’ / ‘E’	9.4	18.8
Palm Tree	Palm tree	100.0	Monster	12.5	87.5	**Palm Tree / Tree**	**59.4**	Saw Blade	15.6	43.8	Cactus	18.8	Face	15.6	3.1
Rhino	Rhino	100.0	Ghost / Monster	18.8	81.3	Dinosaur	18.8	Dog	18.8	0.0	Person	28.1	Gargoyle	21.9	6.3
Elephant	Elephant	96.9	Landscape	9.4	87.5	**Elephant**	**90.6**	Person	15.6	75.0	**Elephant**	**50.0**	Mouth	9.4	40.6
Eagle	Eagle	96.9	Landscape	9.4	87.5	**Bird**	**18.8**	Face	34.4	-15.6	Man with Hat	37.5	Person	9.4	28.1
Duck	Duck	96.9	Tree	15.6	81.3	**Duck**	**75.0**	Cliff	9.4	65.6	Person	15.6	Seahorse	40.6	-25.0
Guitar	Guitar	96.9	Dock	6.3	90.6	Chess Piece	15.6	**Guitar**	**6.3**	9.4	Cloud	9.4	Gun	6.3	3.1
Hand	Hand	96.9	Waves	9.4	87.5	**Fingers / Hand**	**84.4**	Bird	28.1	56.3	**Fingers**	**56.3**	Claw	15.6	40.6
Train	Train	96.9	Faucet	18.8	78.1	Person	50.0	Gun	25.0	25.0	Faucet	21.9	Face	25.0	-3.1
Mickey Mouse*	Mickey Mouse	96.9	Waves	6.3	90.6	**Mickey Mouse**	**34.4**	Landscape	9.4	25.0	Clown	25.0	Knife	6.3	18.8
Trumpet	Trumpet	96.9	**Instrument**	**15.6**	81.3	**Instrument**	**81.3**	Guitar	21.9	59.4	**Instrument**	**56.3**	**Instrument**	**37.5**	18.8
Boot	Boot	93.8	Face	37.5	56.3	**Shoe**	**56.3**	Mouth	12.5	43.8	Mouth	12.5	Lips	9.4	3.1
Flower	Flower	93.8	Person	6.3	87.5	**Flower**	**31.3**	Rhino	6.3	25.0	**Plant**	**50.0**	Leaf	9.4	40.6
Owl	Owl	93.8	Wave	6.3	87.5	**Bird**	**40.6**	Person	12.5	28.1	**Bird**	**50.0**	Monster	18.8	31.3
Pineapple	Pineapple	93.8	Wave	6.3	87.5	Clouds	21.9	Leaf	12.5	9.4	Berries	18.8	Leaf	31.3	-12.5
Foot	Foot	93.8	Stalactites / Icicles	15.6	78.1	Baby	34.4	Scarf	12.5	21.9	Hair	12.5	Plant	18.8	-6.3
Butterfly	Butterfly	93.8	Mountainside	9.4	84.4	**Butterfly / Wings**	**37.5**	Brass Instrument	15.6	21.9	**Butterfly**	**15.6**	Trumpet	12.5	3.1
House	House	93.8	Steam Whistle	15.6	78.1	Nose	12.5	Diving Board	12.5	0.0	Shelf	15.6	Heartbeat Signal	6.3	9.4
Face	Face	90.6	Vase	15.6	75.0	**Face**	**59.4**	Vase	18.8	40.6	**Face**	**25.0**	**Face**	**71.9**	-46.9
Faucet	Faucet	90.6	Face	18.8	71.9	**Faucet**	**81.3**	Puzzle Piece	12.5	68.8	**Faucet**	**53.1**	Puzzle Piece	12.5	40.6
Snowman	Snowman	90.6	Waves	6.3	84.4	Bird	28.1	Bridge	6.3	21.9	Cloud	28.1	Waves	6.3	21.9
Toilet	Toilet	90.6	Mouth	9.4	81.3	Sink	34.4	Building	9.4	25.0	Shoe	59.4	Desk	15.6	43.8
Tree	Tree	90.6	Rock formation	28.1	62.5	Mountain	21.9	Mountain	18.8	3.1	Mountains	21.9	Mountain	25.0	-3.1
Watering Can	Watering Can	90.6	Person	9.4	81.3	**Watering Can**	**50.0**	Tool	9.4	40.6	**Spout**	**59.4**	Mouth	15.6	43.8
Umbrella	Umbrella	90.6	Cat	21.9	68.8	**Umbrella**	**87.5**	Ocean	12.5	75.0	**Umbrella**	**68.8**	Mouth	18.8	50.0
Woman	Woman	87.5	Waves	9.4	78.1	Lamp	43.8	Plant	12.5	30.5	Vase	15.6	**Person**	**12.5**	3.1
Anchor	Anchor	84.4	Puzzle Piece	12.5	71.9	Tree	28.1	Mouth	25.0	3.1	Tree	43.8	Face	12.5	31.3
Axe	Axe	84.4	Hand/Fingers	15.6	68.8	**Axe**	**34.4**	Anvil	15.6	18.8	**Axe**	**53.1**	Corkscrew	18.8	34.4
Dog	Dog	84.4	Face	12.5	71.9	Mountain	15.6	Mountain	25.0	-9.4	Person	43.8	Mountain	25.0	18.8
Seahorse	Seahorse	84.4	Tree	12.5	71.9	**Seahorse**	**25.0**	Winged Animal	21.9	3.1	Praying People	28.1	Dragon	18.8	9.4
Cow	Cow	81.3	Face	12.5	68.8	Mouth	37.5	Dog	12.5	25.0	Mouth	18.8	Mouth	6.3	12.5
Lightbulb	Lightbulb	81.3	Vase	15.6	65.6	Vase	46.9	Knife	15.6	31.3	Breasts	12.5	Vase	18.8	-6.3
Bell	Bell	78.1	Vase / Urn	18.8	59.4	Lamp	68.8	Person	15.6	53.1	Lamp	15.6	Lamp	25.0	-9.4
Fire Hydrant	Fire Hydrant	78.1	Traffic Light	9.4	68.8	Smokestack	12.5	Building	31.3	-18.8	Key	25.0	Building	28.1	-3.1
Teapot	Teapot	75.0	Bearded Man	21.9	53.1	Tree	21.9	Face	6.3	15.6	Person / Child	53.1	Mountain	18.8	34.4
Wine Glass	Wine Glass	75.0	Cleaver	9.4	65.6	**Wine Glass**	**37.5**	Wood	12.5	25.0	Top Hat	78.1	Mouth	18.8	59.4
Maple Leaf	Maple leaf	71.9	Face	21.9	50.0	Crystals	9.4	Mountain	21.9	-12.5	**Leaf**	**9.4**	Cityscape	15.6	-6.3
Pig	Pig	71.9	Canyon	6.3	65.6	Alien	12.5	**Pig**	**18.8**	-6.3	Plant	15.6	**Pig**	**56.3**	-40.6
Spray Bottle	Spray Bottle	68.8	Person	15.6	53.1	Water Fountain	34.4	Cartoon / Face	34.4	0.0	Faucet	21.9	Face	34.4	-12.5
Grapes	Grapes	65.6	Stairs	6.3	59.4	Clouds	56.3	Tree / Leaf	46.9	9.4	Clouds	75.0	Leaf	59.4	15.6
Turtle	Turtle	56.3	Cave	6.3	50.0	Rabbit	34.4	Knife	9.4	25.0	**Turtle**	**40.6**	Seahorse	9.4	31.3
Wrench	Wrench	53.1	Face	18.8	34.4	Rhino	9.4	Tree	9.4	0.0	Tree	43.8	Face	21.9	21.9
Bottle	Bottle	40.6	Column	15.6	25.0	Stove/Furnace	18.8	Glass	18.8	0.0	Lamp post	25.0	**Bottle**	**28.1**	-3.1
Bear	Bear	37.5	Mountains	9.4	28.1	Feet	9.4	Cityscape	12.5	-3.1	Crowd	9.4	Mountainside	9.4	0.0
*Rabbit*	*Lips*	***34*.*4***	*Face*	*25*.*0*	*9*.*4*	*Face*	*18*.*8*	*Waves*	*6*.*3*	*12*.*5*	*Person*	*18*.*8*	*Waves*	*9*.*4*	*9*.*4*
*Jet*	*Man w long face*	*34*.*4*	*Gun*	*12*.*5*	*21*.*9*	*Nose*	*25*.*0*	*Mouth*	*12*.*5*	*12*.*5*	***Airplane***	*15*.*6*	*Tree*	*12*.*5*	*3*.*1*
*Apple*	*Apple*	*31*.*3*	*Neck*	*31*.*3*	*0*.*0*	*Chin*	*18*.*8*	*Hand*	*9*.*4*	*9*.*4*	*Nose*	*12*.*5*	*Waves*	*9*.*4*	*3*.*1*
*Pear*	*Guitar*	*78*.*1*	*Waves*	*6*.*3*	*71*.*9*	*Woman*	*31*.*3*	*Waves*	*18*.*8*	*12*.*5*	*Female Body*	*46*.*9*	*Stringed Instrument*	*18*.*8*	*28*.*1*
**Mean (48)**		**81.3**		**14.0**	**67.3**		**37.9**		**16.2**	**21.7**		**32.5**		**19.9**	**12.6**
*Mean (44)*		*84*.*7*		*13*.*6*	*71*.*1*		*39*.*3*		*16*.*6*	*22*.*6*		*33*.*3*		*20*.*6*	*12*.*7*

The five columns under each type list (1–2) the interpretation with the highest inter-subject agreement for the critical side (Critical) and the percentage agreement for that interpretation, (3–4) the interpretation with the highest inter-subject agreement for the complementary side (Comp) and the percentage agreement for that interpretation, and (5) the critical–complementary difference (Diff). The first column denotes the source object, the object intended to be depicted on the critical side of the border of *Upright Intact* stimuli. Stimuli are ordered from top to bottom by percent inter-subject agreement regarding the interpretation for the critical side of the border of the *Upright Intact* displays. For the four objects listed in italics at the bottom, either the interpretation with the highest inter-subject agreement was different from the source object or the critical–complementary difference was 0. Two means are listed at the bottom of the table; the overall mean is in boldface, and below that, the mean without those four stimuli and their variants is in italics. The interpretations shown in boldface for *Upright Part-Rearranged* and *Inverted Part-Rearranged* stimuli where neither side depicts an intact familiar object are interpretations that match the source object. *Note that the Mickey Mouse stimulus is labelled as “Mickey” in the result and stimulus files.

### Procedure

**Fig 2:**
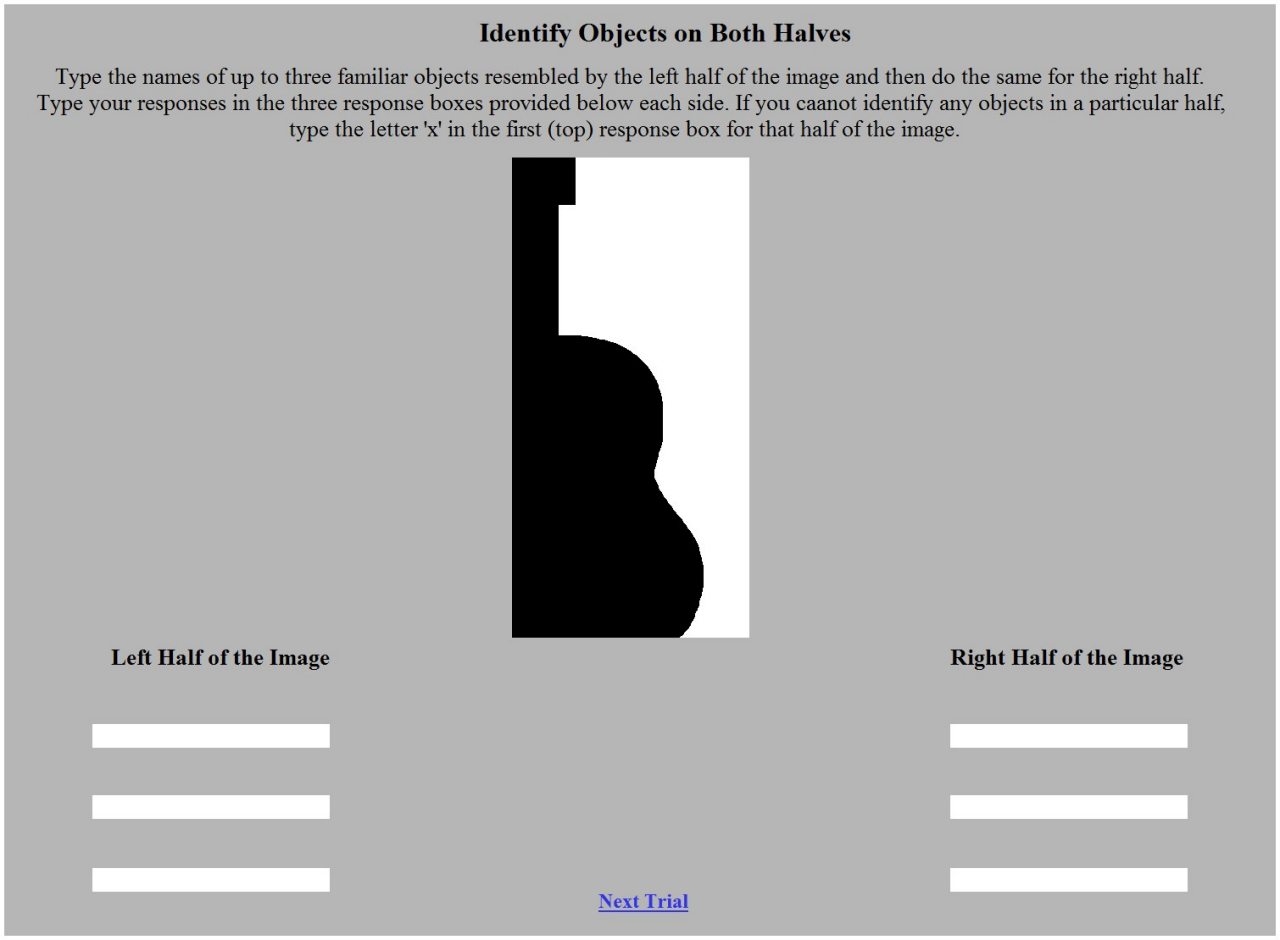
A sample trial. Participants were presented with a bipartite stimulus; here, an *Upright Intact* version of the source stimulus “guitar” sketched in black on the left of the central border. Six response boxes were provided (three per side). Participants used these boxes to list any familiar objects resembled by each side of the stimulus. A button labelled ‘Next Trial’ would lead them to the next trial when they were ready.

Participants could take up to one hour to complete the study. They began by viewing a consent form that was approved by the Human Subjects Protection Program at the University of Arizona. Participants could continue to the rest of the study only after indicating that they had read the consent form and agreed to participate. Next, they viewed a page of instructions. The instructions showed a sample trial (see Fig 2) and informed participants to use the three response boxes on the right and left sides of the screen to list up to three familiar objects resembled by the corresponding regions of the bipartite display. Participants were told they could type an ‘x’ in the top response box if they did not see any familiar objects on that side. Participants could not proceed to the next trial (the next page) without entering something in the top response boxes on the left and right sides. After doing so, they pressed a button to continue.

After the instructions, participants completed 26 trials: 24 trials with bipartite displays and two attention check trials. Of the 24 trials with bipartite displays, eight trials tested each of the three configuration types (*Upright Intact*, *Upright PR*, *Inverted PR*). For each display type, the critical side was equally likely to be black or white, and located on the left or right. On the two attention check trials, the bipartite stimulus was replaced with a white box. The white box contained instructions on how to respond (e.g., “Please write ‘cold’ in the top left and right box”; the words chosen were common words that did not name concrete objects). The attention check trials were included to make sure that participants understood and followed the task. If participants responded incorrectly on the attention check trials, their responses to the bipartite displays were discarded before they were viewed. The trials were presented in a random order. The time to complete each trial was unrestricted.

#### Stimulus presentation programs

Stimulus presentation programs were created as HTML files using Javascript/CSS/HTML and the JQuery Javascript library (version 1.11.3, https://jquery.com), and copied as source code into AMT. Instructions and stimuli were hosted on Imgur (https://imgur.com); their URLs were referenced by the programs. There were two sets of programs, A and B, each presenting 24 of the 48 source stimuli. Within each set there were 12 programs. All three display types (*Upright Intact*, *Upright PR*, and *Inverted PR*) were tested in each program (8 of each type). In a given program, a source stimulus was presented in only one of the three types of displays. Participants never viewed the same source stimulus in any display type more than once. Across the 12 programs in each set, a given source stimulus was tested in all display types. Within and across programs, black/white contrast and left/right location of the critical sides were balanced. Thus, across the 12 programs in each of the two sets, every stimulus was shown equally often in each of its three display types, and within display type, equally often with the critical side in black/white and on the left/right.

Eligible participants could access only one program per set. Thus, they were never exposed to a given source stimulus in more than one display type. Each program was viewed by 8 participants. Of the 174 participants, 156 completed one program only and provided responses for 8 bipartite stimuli of each type; 18 participants completed two programs (in different sets with no overlap in source stimulus) and provided responses for 16 stimuli of each type. In total, 32 participants provided up to three responses for each of the critical and complementary sides of each source stimulus in each type of display.

## Data analysis

Responses from all the programs were sorted according to source stimulus and display type (*Upright Intact*, *Upright PR*, or *Inverted PR*), and stimulus side (critical or complementary). Responses for each side were collapsed over contrast (black/white) and location relative to the central border (left/right) and compiled across 32 participants (up to 96 responses per side given that participants could make up to three responses per side). Next, scorers cleaned up typing/spelling errors (e.g., consolidating ‘trumpet’ and ‘trumpit’) and grouped responses that seemed to denote similar object categories (e.g., ‘clarinet’ and ‘trumpet’ were grouped into single category response for the “Trumpet” source stimulus). These groupings were the basis for the inter-subject agreement scores (see below). Because participants differed in the level of specificity with which they identified objects resembled by the stimuli, responses made by different subjects were considered the same if they labeled the same basic-level object with a different name. For example, the responses ‘dwelling’ and ‘house’ made by different participants were both taken as evidence that the House source stimulus had been recognized at the basic level. If a single participant made two responses that were synonymous for a given region (e.g., ‘house’ and ‘dwelling’ as two different responses for the critical side of the border of the *Upright Intact* version of the House source stimulus), only one was counted so that participants could not contribute multiple interpretations to a single object category. Each grouping of responses into one object category was initially made by naïve scorers; their groupings were checked and confirmed by a second naïve scorer. Differences were discussed and resolved by the authors.

## Results and discussion

The object category identified by the largest number of participants for a given side of the border of a given stimulus was selected as the best fitting interpretation. The number of participants who made this response was divided by 32 (the maximum number of responses if every participant contributed one response) to determine the percent inter-subject agreement regarding this object category. These inter-subject agreement percentages are shown in Table 1, where the anticipated identities of the 48 source stimuli are listed in the left column and the three variants of these source stimuli–*Upright Intact*, *Upright PR*, and *Inverted PR*–are arranged from left to right with five columns embedded under each type. These five columns list from left to right (1) the object category with the highest inter-subject agreement for the critical side of the central border, (2) the percent inter-subject agreement for that object category, (3) the object category with the highest inter-subject agreement for the complementary side of the central border, (4) the percent inter-subject agreement for that object category, and (5) the difference between the inter-subject agreement percentages for the critical and complementary sides of the border. Across all stimuli and both sides, the inter-subject agreement percentages ranged from 6.3% to 100%.

### *Upright Intact* displays

#### Critical side

When all stimuli were considered, the mean inter-subject agreement for the critical side of the border of *Upright Intact* displays was 81.3%, indicating that on average the critical sides of the borders are good depictions of the source stimuli. In Table 1, the source stimuli are sorted by inter-subject agreement with one exception–the Pear stimulus is listed last because 78.1% of participants misidentified the critical side of the border as a “guitar.” The critical side of the *Upright Intact* Jet display was also misidentified: 34.4% of participants identified it as a “face.” Given that inter-subject agreement was > 90% for the critical sides of source stimuli Guitar and Face, we recommend against using these stimuli, but we list the results here for completeness and to allow individual experimenters to make their own decisions.

We recommend against using two other stimuli as well: the Rabbit source stimulus because the critical side was identified as “lips” by the largest percentage of participants (34.4%) rather than as a rabbit; and the Apple source stimulus because inter-subject agreement was low (31.3%) and not different from the inter-subject agreement regarding the complementary side. We list the mean inter-subject agreement without these four stimuli in italics below the overall mean.

#### Complementary side

The data indicate that the complementary sides of the borders of *Upright Intact* displays are not good depictions of well-known objects. Mean inter-subject agreement regarding objects denoted on the complementary side of all 48 stimuli is low: 14.0%. We had originally intended to use only bipartite displays in which participants indicated that the complementary side did not resemble anything familiar in order to test the role of familiar configuration in the absence of any other figural prior, as has been done for other figural priors. In our early work, we found that finding complementary sides for which no participants agreed on an interpretation was nearly impossible. Accordingly, in previous research, we had instead set an upper cut-off of 23% inter-subject agreement for complementary sides, reasoning that with such low inter-subject agreement the complementary side of the central border of *Upright Intact* displays could be taken to depict nominally “novel” objects [19]. We no longer set an a priori cut-off for the complementary sides of the displays, although individual experimenters may choose to do so: Inter-subject agreement for the object category resembled by the complementary side of the border of four of the 48 *Upright Intact* displays was > 25% (source stimuli: Apple, Boot, Tree, and Rabbit).

We note that the interpretations listed for the complementary side of the border of 15 of the *Upright Intact* displays were landscape features rather than objects: see responses of “waves,” “mountainside,” “rock formation,” building,” “canyon,” “cave,” and “landscape,” (only “building” and “rock formation” generated > 25% agreement). It is currently unknown whether past experience with landscape features influences figure assignment. The present set of stimuli allows a test of that hypothesis.

#### Critical—Complementary difference

The difference between the inter-subject agreement for the critical and complementary sides of the border shown in the fifth column under *Upright Intact* displays was large: 67.3% on average.

#### Summary for *Upright Intact* displays

For *Upright Intact* displays the mean inter-subject agreement is 81.3% for the critical side (median = 90.6%; range = 31.3%–100%), 14.0% for the complementary side (median = 12.5%; range = 6.3%–37.5%), and the mean *critical–complementary difference* was 67.3%, (median = 71.9%; range 0–90.6%). Thus, for the majority of the *Upright Intact* displays in the OMEFA-II set the central border sketches a good depiction of a common object on the critical side and a poor depiction on the complementary side. The average critical–complementary difference was large, suggesting that the critical side would tend to win the competition for figure assignment.

### *Upright PR* displays

#### Critical side

When all stimuli were considered, the mean inter-subject agreement for the critical side of *Upright PR* displays was 37.9%. This percentage indicates substantially lower inter-subject agreement than for the *Upright Intact* displays which we take as evidence that, as a set, the *Upright PR* displays are less likely to activate memory traces of well-known objects. For the critical sides of 18 of the *Upright PR* stimuli, however, the highest inter-subject agreement was for the same object category as for the critical side of the *Upright Intact* displays (see interpretations in boldface). These responses are probably based on identification of a distinctive part (e.g., the elephant’s trunk). The mean inter-subject agreement for these 18 stimuli (54.7%) was substantially lower than for the corresponding *Upright Intact* stimuli (92.4%) yet higher than for the remaining 30 stimuli (27.9%) for which inter-subject agreement indicated that they resembled objects other than the source objects. For 15 of these remaining displays, inter-subject agreement for the critical side was > 25% (source stimuli as listed from top to bottom in Table 1: lamp, train, foot, snowman, toilet, woman, cow, anchor, light bulb, bell, spray bottle, grapes, turtle, jet, and pear). The probability of perceiving the figure on the critical side of the border may be higher for these displays than for displays with lower inter-subject agreement for the critical side.

#### Complementary side

Mean inter-subject agreement regarding the category of the objects resembled by the complementary sides of *Upright PR* displays was low (16.2%); lower than for the critical sides of *Upright PR* stimuli that didn’t support identification based on diagnostic parts and approximately the same as for the complementary side of the *Upright Intact* displays (14.0%). Inter-subject agreement was > 25% for the complementary sides of eight of the source stimuli (Eagle, Hand, Train, Anchor, Dog, Fire Hydrant, Spray Bottle, and Grapes). Two of these interpretations (“mountain” and “building”) were landscape features rather than objects per se.

#### Critical—Complementary differences

The mean difference between the inter-subject agreement for the critical and complementary sides observed for *Upright PR* displays was 21.7%, quite a bit smaller than for *Upright Intact* displays, primarily because inter-subject agreement was low regarding objects resembled by the critical side. The c*ritical–complementary differences* were negative for five stimuli (range: –18.8 to –3.1). Most of these negative differences were small; three were obtained when the inter-subject agreement for the complementary side was a landscape feature, and one may be an instance of pareidolia. It is not clear whether the activation of past experience is weaker for pareidolia interpretations than for other interpretations. Note, however that competition for figural status may favor the complementary side as figure in stimuli with a negative c*ritical–complementary difference*. Therefore, the sign of the c*ritical–complementary difference* should be considered when comparing performance with *Upright PR* displays and *Upright Intact* displays.

#### Summary for *Upright PR* displays

For the *Upright PR* displays in the OMEFA-II set, the mean inter-subject agreement was 37.9% for the critical side (median = 34.4%; range = 9.4%–90.6%); 16.2% for the complementary side (median = 12.5%; range = 6.3%–46.6%) and the mean *critical–complementary difference* was 21.7%, (median = 21.9%; range -18.8%—+75.0%). As manifested by participants’ explicit responses, overall, the critical sides of the *Upright PR* displays are less likely to activate traces of previously seen objects. Inter-subject agreement was higher for some of the displays, however, perhaps because of the presence of distinctive parts. The normative data provided here can be used to investigate whether familiar parts play a larger role in object detection for the 18 *Upright PR* displays that supported identification of the source object under the long exposure durations tested here. Such tests would be informative for both participants with intact brains as well as for participants with damage to the PRC of the MTL who have shown effects of familiar parts that are absent when the PRC is intact [18]. Previous tests comparing performance with *Upright Intact* versus *Upright PR* displays included only five of the displays that were found to support the highest agreement for the source object in the present study [18, 32, 33]. The OMEFA-II set can provide a sensitive test of the role of distinctive parts in figure assignment in participants with intact brains and with PRC damage. It would also be interesting to compare PRC activation for *Upright Intact* versus *Upright PR* displays in the larger OMEFA-II set presented here as a function of whether the latter produced the highest inter-subject agreement for the source object.

### *Inverted PR* displays

#### Critical side

The mean inter-subject agreement regarding well known objects resembled by the critical side of the border of *Inverted PR* displays (32.5%) was substantially lower than for the *Upright Intact* displays but only slightly lower than for the *Upright PR* displays. We take these data as evidence that the critical sides of these stimuli do not highly activate past experience with well-known objects. For 14 of the 48 *Inverted PR* stimuli, however, the largest percentage of reports was that the critical side resembled the source object. Once again, we hypothesize that this high inter-subject agreement is based on the identification of distinctive parts: 11 of these 14 displays are a subset of the 18 *Upright PR* displays for which participants agreed that the critical side of the border resembled the source object. The mean inter-subject agreement for these 11 *Inverted PR* displays (48.9%) was lower than for the corresponding *Upright PR* displays (61.6%). Thus, under the long exposure conditions of this norming study, inverted and upright diagnostic parts are approximately equally likely to support object identification. It remains to be tested whether these *Inverted PR* displays support familiarity effects on figure assignment. Participants with damage to the PRC of the MTL showed familiarity effects with *Upright PR* displays but not with *Inverted PR* displays [18], but those experiments included only five of the 14 *Inverted PR* displays that were identified as the source object in the present study. Perhaps past experience with familiar parts is orientation-dependent, as past experience with familiar configurations is ([18, 33, 41]; but see [42]). Although the long exposures of the current study can support part-based identification, effects on figure assignment may be absent because past experience cannot be activated as quickly for inverted parts as for upright parts (similar to intact displays). With the OMEFA-II set, this hypothesis can be tested more sensitively.

#### Complementary side

Inter-subject agreement regarding the object category resembled by the complementary side of the border of the 48 stimuli in the new OMEFA-II set was 19.9%. For five of the displays, the highest inter-subject agreement was for the source object (source objects: Face, Woman, Trumpet, Bottle, and Pig). The complementary side of the *Inverted PR* Pig stimulus was interpreted as a pig by 56.3% of observers. This was very high, and upon inspecting the stimulus after observing this result, we could see that a configuration of parts (rather than a single pig-like part) probably supported this interpretation. This was not detected in the creation of the stimulus and demonstrates the importance of measuring interpretations from naïve observers. The *Inverted PR* Pig stimulus should be used with caution. For nine other displays, inter-subject agreement that the complementary side of the border denoted a different object was > 25% (Duck, Train, Pineapple, Tree, Dog, Bell, Fire Hydrant, Spray Bottle, and Grapes). Three of these interpretations were landscape features rather than objects, one was an instance of pareidolia, and two named a single part of the novel configuration created by rearranging the parts of the source object (e.g., “leaf” for Grapes and Pineapple).

#### Critical—Complementary difference

The mean difference between the inter-subject agreement for the critical and complementary sides of *Inverted PR* displays was 12.6%, smaller than for the *Upright PR* displays. The smaller difference was obtained because inter-subject agreement was both lower for the critical side and higher for the complementary side. The *critical–complementary differences* were negative for 13 stimuli (range: –46.9% to –3.1%). The largest negative difference was obtained when the inter-subject agreement for the complementary side was for “face,” which may be a manifestation of pareidolia. It is not clear whether pareidolia manifests based on fast or slow activation of past experience. Results obtained with these stimuli should be examined to determine whether the figure is more likely to be perceived on the complementary side of stimuli with negative *critical–complementary differences*.

#### Summary for *Inverted PR* displays

For the *Inverted PR* displays in the OMEFA-II set, the mean inter-subject agreement was 32.5% for the critical side (median = 25.0%; range = 9.4%–78.1%); 19.9% for the complementary side (median = 17.2%; range = 6.3%–71.9%) and the mean *critical–complementary difference* was 12.6%, (median = 9.4%; range = -40.6%–59.4%).

## Concluding thoughts

We obtained normative data regarding familiar objects resembled by (denoted by) both the critical and complementary sides of 144 high resolution stimuli–the OMEFA-II set. The OMEFA-II set comprises three different types of bipartite displays: *Upright Intact* displays (N = 48; 96 sides), *Upright PR* displays (N = 48; 96 sides), and *Inverted PR* displays (N = 48; 96 sides). The normative data presented here are contemporary and comprehensive, including inter-subject agreement for both critical and complementary sides of the three types of bipartite displays and the difference between inter-subject agreement for the two sides. Stimulus characteristics for the OMEFA-II displays are also reported in S1 Appendix. The OMEFA-II set is larger and higher in resolution than the original OMEFA set and the normative data presented here are more comprehensive than the data previously available for the original set. The OMEFA-II displays will be valuable for experiments investigating questions concerning (a) how parts versus wholes activate past experience before object detection, (b) which brain areas/networks are involved in high-level influences on object detection, and (c) the role of competition in object detection, among others. These stimuli will be useful for tests of participants with intact brains as well as damaged brains, for experiments using brief exposures as well as the longer exposures required for brain-damaged participants.

The inter-subject agreement reported here is one way to operationalize familiar configurations. Behavioral measures such as the probability of perceiving the figure on the critical side of the border, event-related potentials (ERPs), and the blood oxygen dependent (BOLD) response in fMRI experiments, perhaps in combination with multi-voxel pattern analysis (MVPA), may also quantify activation of past experience. Correlating the data presented in Table 1 with other indices such as these may be fruitful in understanding object perception in general, figure assignment in particular, and any underlying competition between objects that might be perceived on opposite sides of a border.

In addition to inter-subject agreement for the critical and complementary sides of the border individually, we report the difference in inter-subject agreement regarding the objects sketched on the critical versus the complementary sides of the border. On current inhibitory competition accounts of figure assignment (e.g., [43, 44]), this difference may better predict whether a figure will be perceived on the critical side of a border than the inter-subject agreement regarding the critical side alone. The comprehensive set of norms presented here allows future experiments to test which is the better predictor.

Although inter-subject agreement is informative about which common objects were activated, it cannot assess how quickly they were activated. In previous research, substantially larger effects of familiar configuration were found for *Upright* than *Inverted Intact* displays (e.g., [2, 14, 19, 45]). This orientation-dependent difference has been attributed to the time required for evidence to accumulate in neural populations activated by the familiar object (longer for *Upright* than *Inverted* displays; [17]). The orientation-dependency of the familiar configuration prior has been taken to indicate that priors for figure assignment must be available quickly in order to influence figure assignment (for review see [5]). Indeed, once the critical sides of *Inverted Intact* displays are perceived as figures, the familiar objects they portray can often be identified. (This is why we did not obtain norms for the critical side of *Inverted Intact* displays.) Nevertheless, knowing that critical sides depict inverted familiar objects does not increase the likelihood of seeing the figure on the side where an inverted version of the intact object is sketched [14].

For some of the *Upright* and *Inverted Part-Rearranged* displays, sizeable inter-subject agreement seemed to be based on diagnostic parts. In future research it will be interesting to test whether access to object categories via diagnostic parts as evidenced by these explicit responses generated while the stimuli were exposed for long durations is sufficient for past experience effects on figure assignment. Given the large set normed here this can be done for both *Upright* and *Inverted PR* displays by comparing performance with the subsets of displays for which the largest percentage of participants did versus did not identify the source stimulus. Previous studies have shown that for both young and old participants the critical side of the border is substantially and significantly less likely to be perceived as the figure in *Upright PR* displays than *Upright Intact* displays (e.g., [14, 18, 19, 22, 29]), yet none of those experiments used the large set of stimuli normed here that affords a sensitive analysis of differences within the set of *Upright PR* displays based on whether or not distinctive parts supported identification of the source stimulus.

Some of the interpretations that garnered >25% agreement were landscape features rather than objects. A small percentage of similar responses was observed in previous norming studies, but they did not exceed 25% agreement. It could be interesting to test whether, for an equivalent level of inter-subject agreement, landscape features and concrete objects are equivalent priors for figure assignment.

## Summary

We present normative data obtained for an expanded set of bipartite stimuli–the OMEFA-II stimulus set–that is well-suited for assessing high-level influences on figure assignment, an essential component of object perception. The bipartite stimuli are divided into two equal area regions by a central border. Normative data were obtained by presenting the bipartite stimuli to AMT participants who were asked to identify any familiar objects sketched by central border on both a critical side and a complementary side. The critical side depicted either an intact version of an upright familiar object (*Upright Intact* displays), or a part-rearranged version in an upright or inverted orientation (*Upright PR* and *Inverted PR* displays respectively). The stimuli (including *Upright Inverted* displays), as well as Excel files of Table 1, S1 Appendix, the AMT data sorted by stimulus type (and within stimulus type by critical and complementary side), and the full data set are available online (https://osf.io/j9kz2/).

## Supporting information

S1 Appendix (DOCX)
