# Supplementary material for: Normative data for an expanded set of stimuli for testing high-level influences on object perception: OMEFA-II
Source: PLoS One. 2020 Aug 14;15(8):e0224471. doi: 10.1371/journal.pone.0224471 (PMC7428090; doi:10.1371/journal.pone.0224471)
Supplement: S1 Appendix — (DOCX) [file pone.0224471.s001.docx]

S1 Appendix. Image statistics for bipartite images. Statistics are shown for *Intact* and *Part-Rearranged* displays. The statistics are the same for both *Upright* and *Inverted* orientations. Category denotes whether the source object is Natural (N) or Artificial (A); * = ambiguous. “Area (px)” is the total number of pixels in the display. “% Area Crit Side” is the percentage of pixels on the critical side of the border. The percentage of pixels on the complementary side of the border is (1 – “% Area Crit Side”). “Border Length” is the length of the central border in pixels calculated using the bwperim function in MATLAB (2016b; MathWorks, Natick, MA).

|  |  | **Intact** | | | **Part-Rearranged** | | |
| --- | --- | --- | --- | --- | --- | --- | --- |
| Source Object | Category | Area (px) | % Area Crit Side | Border Length (px) | Area (px) | % Area Crit Side | Border Length (px) |
| anchor | A | 70315 | 49.82 | 744 | 69629 | 50.10 | 584 |
| apple | N | 66542 | 49.97 | 451 | 69629 | 49.90 | 451 |
| axe | A | 49392 | 49.92 | 618 | 49392 | 49.79 | 604 |
| bear | N | 48363 | 50.04 | 630 | 50421 | 50.00 | 665 |
| bell | A | 59339 | 50.19 | 536 | 59339 | 50.10 | 485 |
| boot | A | 78890 | 50.04 | 565 | 78890 | 49.87 | 577 |
| bottle | A | 70315 | 49.66 | 368 | 70315 | 50.18 | 374 |
| butterfly | N | 118678 | 49.94 | 1043 | 118678 | 50.06 | 1013 |
| cow | N | 82320 | 49.95 | 623 | 80262 | 50.03 | 572 |
| dog | N | 79233 | 50.01 | 581 | 86436 | 49.90 | 589 |
| duck | N | 73402 | 49.92 | 535 | 73402 | 50.05 | 536 |
| eagle | N | 71687 | 50.06 | 454 | 71687 | 50.13 | 458 |
| elephant | N | 102900 | 49.93 | 809 | 102900 | 50.11 | 778 |
| face | N | 79233 | 50.02 | 415 | 79233 | 49.89 | 415 |
| faucet | A | 89523 | 50.06 | 728 | 89523 | 50.11 | 728 |
| fire hydrant | A | 81291 | 49.90 | 479 | 81291 | 50.13 | 450 |
| flower | N | 80262 | 49.94 | 945 | 80262 | 50.09 | 884 |
| foot | N | 62083 | 49.99 | 606 | 62083 | 49.91 | 603 |
| grapes | N | 68257 | 50.07 | 552 | 68257 | 50.14 | 540 |
| guitar | A | 57967 | 50.09 | 395 | 58310 | 50.02 | 384 |
| hands | N | 70658 | 50.04 | 794 | 70658 | 50.01 | 771 |
| house | A | 84035 | 50.00 | 470 | 84035 | 50.00 | 541 |
| jet | A | 45962 | 50.03 | 530 | 45619 | 50.08 | 533 |
| lamp | A | 58653 | 49.95 | 474 | 58653 | 47.86 | 458 |

(continued on next page)

|  |  | **Intact** | | | **Part-Rearranged** | | |
| --- | --- | --- | --- | --- | --- | --- | --- |
| Source Object | Category | Area (px) | % Area Crit Side | Border Length (px) | Area (px) | % Area Crit Side | Border Length (px) |
| lightbulb | A | 72030 | 50.06 | 365 | 72030 | 51.89 | 370 |
| maple leaf | N | 84721 | 50.07 | 692 | 84721 | 49.87 | 571 |
| mickey mouse | A* | 62426 | 50.00 | 613 | 62426 | 49.98 | 579 |
| owl | N | 60711 | 49.96 | 649 | 60711 | 50.15 | 631 |
| palm tree | N | 65170 | 49.86 | 739 | 65170 | 49.81 | 732 |
| pear | N | 56252 | 50.05 | 363 | 56252 | 49.78 | 364 |
| pig | N | 73059 | 50.00 | 583 | 72716 | 49.91 | 585 |
| pineapple | N | 79576 | 50.08 | 546 | 79576 | 49.90 | 543 |
| rabbit | N | 47334 | 50.16 | 507 | 47334 | 50.02 | 503 |
| rhino | N | 120050 | 50.02 | 846 | 120050 | 49.94 | 848 |
| seahorse | N | 106673 | 49.96 | 612 | 106673 | 49.93 | 615 |
| snowman | A* | 54537 | 49.94 | 441 | 54537 | 49.97 | 436 |
| spray bottle | A | 40474 | 50.08 | 486 | 40474 | 49.94 | 486 |
| teapot | A | 61397 | 50.02 | 524 | 61397 | 50.26 | 508 |
| toilet | A | 91924 | 50.06 | 633 | 95697 | 49.98 | 622 |
| train | A | 85750 | 49.93 | 612 | 85750 | 49.88 | 619 |
| tree | N | 83349 | 49.90 | 546 | 83006 | 50.02 | 440 |
| trumpet | A | 39445 | 49.99 | 548 | 39445 | 49.96 | 550 |
| turtle | N | 74088 | 49.99 | 799 | 74088 | 50.13 | 800 |
| umbrella | A | 92953 | 50.03 | 651 | 99813 | 50.06 | 652 |
| watering can | A | 105987 | 49.92 | 698 | 105987 | 50.04 | 700 |
| wine glass | A | 50764 | 50.08 | 414 | 50764 | 50.24 | 482 |
| woman | N | 38073 | 50.06 | 415 | 38416 | 49.88 | 393 |
| wrench | A | 76489 | 49.97 | 490 | 76489 | 49.84 | 488 |
| Means |  | 72344.4 | 49.99 | 585.8 | 72758.9 | 50.00 | 573.1 |
